# Building Research Capacity With Older Adults: The Collective of Older Adult Researchers (COAR) Initiative

**DOI:** 10.1093/geroni/igaf122.340

**Published:** 2025-12-31

**Authors:** Rebecca White, Atiya Mahmood, Michael Volker, Mei Lan Fang

**Affiliations:** Simon Fraser University, Vancouver, British Columbia, Canada; Simon Fraser University, Vancouver, British Columbia, Canada; 411 Seniors Centre Society, Vancouver, British Columbia, Canada; Simon Fraser University, Vancouver, British Columbia, Canada

## Abstract

Developing the capacity of older adults to meaningfully engage in research is an increasingly recognized priority in the field of aging. However, practical frameworks for effectively fostering research capacity through collaborative partnerships with older adult community members remain limited. This paper introduces the Collective of Older Adult Researchers (COAR) project, an innovative intergenerational initiative between 411 Seniors Centre Society and Simon Fraser University (Vancouver, Canada). Designed to advance the vision of community research hubs within third places that serve older adults, this initiative prioritizes key aging-related issues while increasing older adults’ direct involvement in research. Through a series of three interactive 2.5-hour workshops, participants engaged in a blend of research methodology presentations and hands-on activities conducted in community spaces. Older adults (n = 10) were paired with graduate students (n = 10) to co-develop and implement a practical research project. A reflexive analysis of the process, conducted in collaboration with older adult co-researchers, identified four key themes that offer insights for strengthening this model and informing broader implementation: (1) Engaging Community Partners in Workshop Design, (2) Fostering Intergenerational Collaboration, (3) Prioritizing Informal Collaborative Dialogue, and (4) Building Pathways for Sustainable Change. Findings highlight the potential of community-based research training to equip older adults with the knowledge and skills needed to become equitable research partners. However, further exploration is required to enhance, sustain, and scale these models, which will be investigated through forthcoming community-engaged research collaborations.

